# Crystal structure and fluorescence of 1-[8-phenyl-9-(phenyl­ethyn­yl)-4*H*-benzo[*def*]carbazol-4-yl]ethan-1-one

**DOI:** 10.1107/S2056989022004509

**Published:** 2022-05-13

**Authors:** Huan-Chang Hsiao, Pei-Lin Chen, Shih-Ching Chuang

**Affiliations:** a Department of Applied Chemistry, National Yang Ming Chiao Tung University, Hsinchu 300093, Taiwan; b Department of Chemistry, National Tsing Hua University, Hsinchu 300044, Taiwan; c Department of Applied Chemistry, National Yang Ming Chiao Tung University, Hsinchu 30010, Taiwan; Universidade de Sâo Paulo, Brazil

**Keywords:** crystal structure, benzo[*def*]carbazole, π–π stacking, fluorescence, dual emission

## Abstract

The crystal of the title compound, C_60_H_38_N_2_O_2_, which crystallizes in the monoclinic space group *P*2_1_/*n*, was obtained by thermal evaporation of a 1:1 di­chloro­methane and hexa­nes solution of the pure compound. The crystal structure is stabilized by π–π inter­actions between benzo[*def*]carbazole moieties and carbon­yl–carbonyl inter­actions between the two acetyl groups.

## Abstract 2

A dual emissive fluorescent substituted benzo[*def*]carbazole was obtained through C—H bond activation catalysed by Pd(OAc)_2_. It crystallizes in the monoclinic space group *P*2_1_/*n*.

## 1. Chemical context

For recent background literature on the chemistry of related carbazole-derived compounds and their applications, including syntheses of bioactive carbazoles, see: (Chakraborty *et al.*, 1965 ▸; Bondock *et al.*, 2019 ▸) and references cited therein. The syntheses of related benzo[*def*]carbazoles are described by Pocock *et al.* (2021 ▸) and Geng *et al.* (2016 ▸). For applications of benzo[*def*]carbazole derivatives, see: Vespa *et al.* (2018 ▸), Atakan & Gunbas (2016 ▸) and Myśliwiec *et al.* (2015 ▸).

The photophysical properties of 4*H*-benzo[*def*]carbazole have been studied over the past few decades (Bender *et al.*, 1964 ▸; Zander *et al.*, 1966 ▸; Favini *et al.*, 1971 ▸; Horaguchi *et al.*, 1980 ▸). The spectra show that the wavelengths of absorption and emission maxima are in the ranges 230–410 nm and 345–520 nm, respectively, at different temperatures and for different solvents. The effect of the solvent on absorption and fluorescence bands as well as comparisons with theoretical expectations have been used to estimate the dipole moment of the first excited state. Geng *et al.* (2016 ▸) reported the optimized geometry, electron-density distributions, and HOMO and LUMO of carbazole and 4*H*-benzo[*def*]carbazole. Changes in the HOMO–LUMO gap (*Eg*) and the design of mol­ecules for material applications can be realized by comparing frontier mol­ecular orbitals, HOMO and LUMO energy levels, and exploring their electron-density maps. 
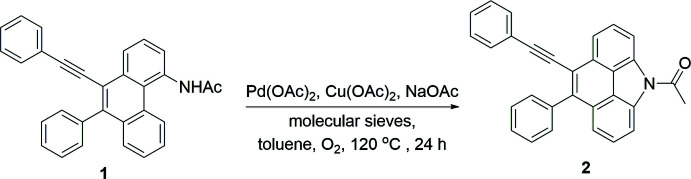


In order to obtain the benzo[*def*]carbazole **2** efficiently, we utilized the pathway through the conversion of di­phenyl­phenanthrene **1** to *N*-acetyl benzo[*def*]carbazole **2**. We obtained *N*-acetyl carbazole **2** in qu­anti­tative yield utilizing Buchwalds’ method by treatment of di­phenyl­phenanthrene **1** as a substrate in the presence of Pd(OAc)_2_ (10 mol %), NaOAc (1.0 equiv.), Cu(OAc)_2_ (2.0 equiv.) and powdered mol­ecular sieves in toluene under oxygen at 393 K for 24 h. Single crystals of **2** were grown from the a mixture of hexa­nes and DCM (*v*/*v* = 1:1) at room temperature by slow thermal evaporation.

## 2. Structural commentary

**Figure 1:**
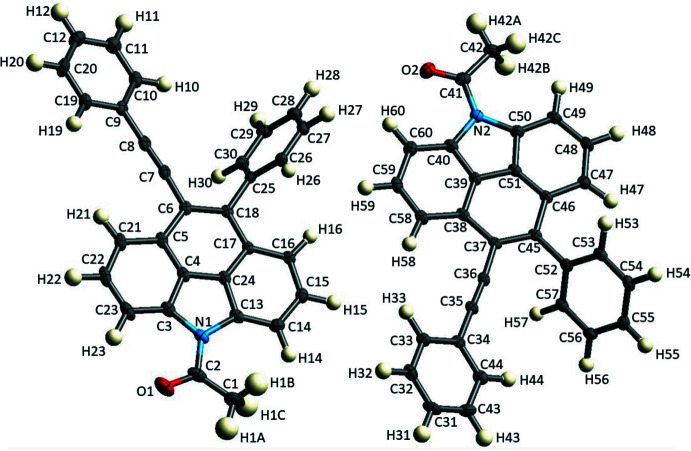
The mol­ecular structure of **2**, showing the atom-labelling scheme and displacement ellipsoids at the 50% probability level.

Compound **2** crystallizes in the monoclinic space group *P*2_1_/*n* with two independent mol­ecules in the asymmetric unit. The atomic labelling scheme is shown in Fig. 1 ▸. The C—C bond lengths are within the expected values known for aromatic systems (Allen *et al.*, 1987 ▸).

In the structure of **2**, both independent conformers occupy their own coordinates in the asymmetric unit, but are in the same configuration. On the other hand, owing to the space group of the title compound, *P*2_1_/*n*, which is centrosymmetric, both mol­ecules will produce two identical configurations that are 180° inverted from each other. In the stereoscopic view, we can observe that the phenyl group and the phenyl alkynyl moiety in the two independent conformers have different dihedral angles with respect to the benzo[*def*]carbazole, which are 22.2 (1), 25.7 (2)° and 50.8 (2), 59.7 (2)°, respectively.

## 3. Supra­molecular features

**Figure 2:**
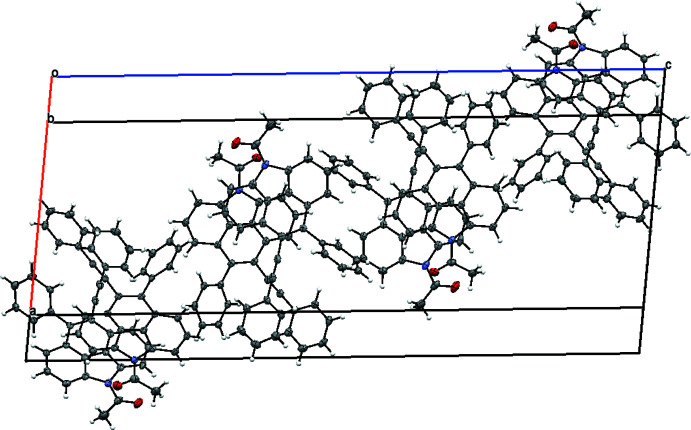
The packing of **2**.

**Figure 3:**
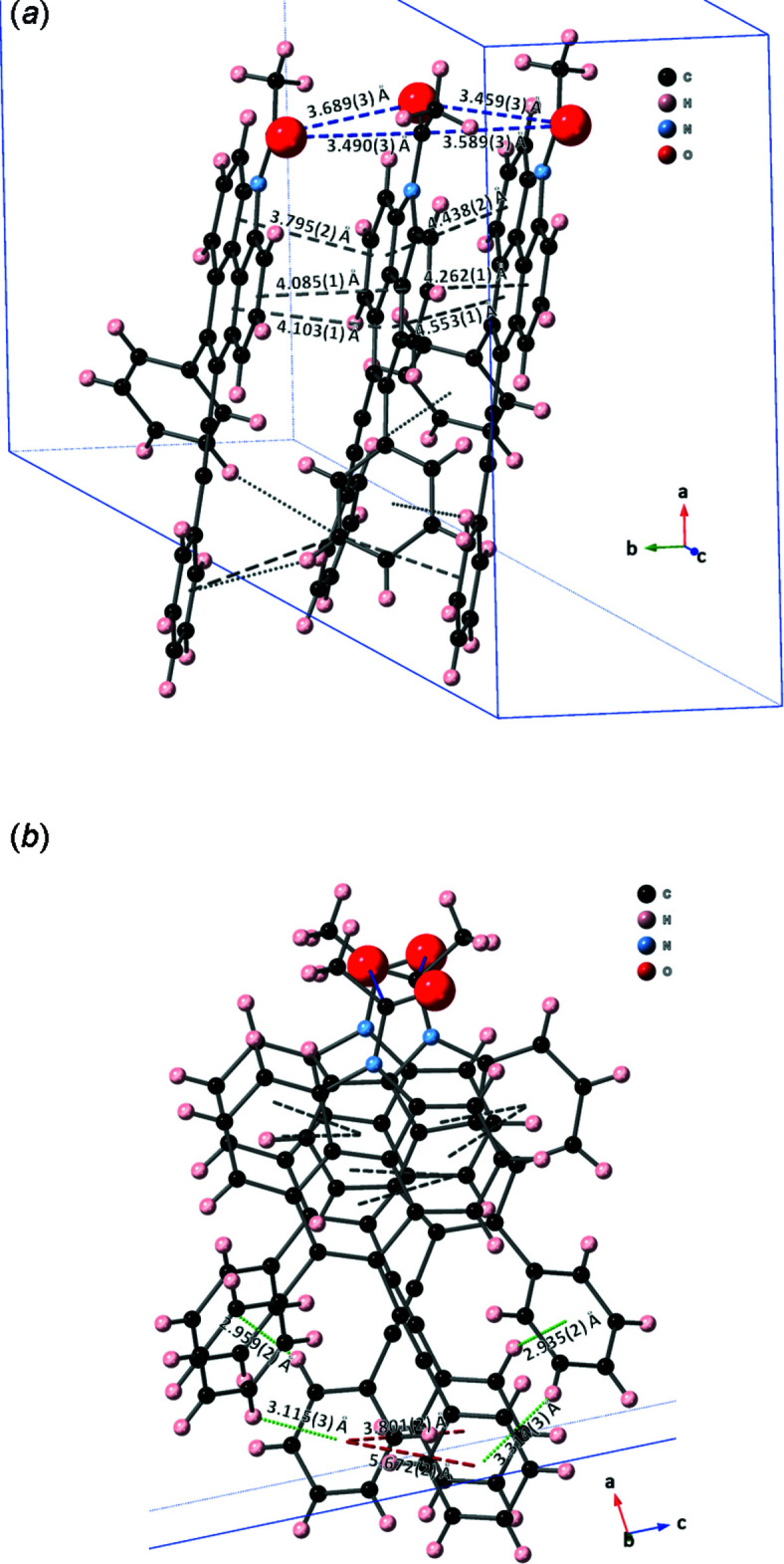
(*a*) A partial view of the crystal packing of **2**. Inter­molecular π–π inter­actions between the benzo[*def*]carbazole moieties and acet­yl–acetyl dipolar inter­actions are depicted by grey and blue dashed lines, respectively. (*b*) A view along the *b* axis of the crystal packing of **2**. The green and red dashed lines represent C—H⋯π and π–π inter­actions, respectively.

In the crystal, there are no classical hydrogen bonds present and the crystal packing of **2** (Fig. 2 ▸) is mainly determined by inter­molecular π–π inter­actions between the benzo[*def*]carbazole moieties with centroid–centroid distances of 3.795 (2) to 4.553 (1) Å (Fig. 3 ▸ *a*, grey dashed line), acet­yl–acetyl dipolar inter­actions of 3.459 (3) to 3.689 (3) Å (Fig. 3 ▸ *a*, blue dashed line), C—H⋯π inter­actions of 2.935 (2) to 3.314 (3) Å (Fig. 3 ▸ *b*, green dashed line), and π–π inter­actions with centroid–centroid distances of 3.801 (2) to 5.672 (2) Å (Fig. 3 ▸ *b*, red dashed lines) between phenyl alkynyl moieties. Specifically, the crystal is stabilized by the phenyl groups of the alkynyl moiety, which inter­acts weakly with each other (Fig. 3 ▸ *b*, red dashed lines) through π–π stacking. Furthermore, the phenyl group also inter­acts with another neighboring phenyl moiety and with the phenyl alkynyl moiety through C—H⋯π inter­actions (Table 1 ▸). In addition, π–π stacking and carbon­yl–carbonyl inter­actions with δC^+^ and δO^−^ between the two acetyl groups are observed. The mol­ecules are ordered into infinite ribbons extending along the [001] direction through alternating inter­molecular C—H⋯π and π–π stacking inter­actions.

## 4. Database survey

A search of the Cambridge Structural Database (WebCSD accessed 21 April 2022; Groom *et al.*, 2016 ▸) results in over a thousand carbazole derivatives of which 45 are derivatives of benzo[*def*]carbazole. Most of the compounds are cyclized with the benzo moiety to the skeleton of benzo[*def*]carbazole. Of these, 31 mol­ecular structures are derivatives of the main structure of phenanthro[1,10,9,8-*cdefg*]carbazole that is commonly used to design functional mol­ecules, such as organic transistors or *n*-doped thermoelectric devices [KUTZUX (Cann *et al.*, 2020 ▸); ZAJMUW (Martell *et al.*, 2021 ▸)], white-light emissive material (ILIGIW; Chatsirisupachai *et al.*, 2021 ▸), *N*-annulated perylene di­imide for stable organic materials with unique optical, electronic, magnetic properties (MEHDUB; Wei *et al.*, 2017 ▸), organic solar cells [NEXKOT (Ma *et al.*, 2018 ▸); EZETOU (Hendsbee *et al.*, 2016 ▸)] and the green solvent processing of organic charge-transporting materials (HUVQEX; Harding *et al.*, 2020 ▸). Other compounds have benzo[*def*]carbazole derivatives as the skeleton, for instance, 4,5-imino­phenanthrene (IMNPHN; Ern *et al.*, 1971 ▸), aka 4*H*-benzo[*def*]carbazole, capped [3]cyclo­(2,6)benzo[*def*]carbazoledi­chloro­methane solvate (ROZQAA; Myśliwiec *et al.*, 2015 ▸), picenoporphyrins [QUQYAC01 (Nath *et al.*, 2003 ▸); QUQYAC (Aihara *et al.*, 2001 ▸)] and 4*H*-naphtho­[1,2,3,4-*def*]carbazole (IWOBEE; Pocock *et al.*, 2021 ▸). In addition, there is no alkynyl phenyl group on C8 and a phenyl group on C9 of the benzo[*def*]carbazole as in the title compound in any structure found in the WebCSD search. The title compound is the only one with an *N*-acetyl group attached to the benzo[*def*]carbazole unit.

## 5. Synthesis and crystallization

To a dried reaction tube, phenanthrene **1** (0.1 mmol), Pd(OAc)_2_ (2.25 mg, 0.01 mmol), Cu(OAc)_2_ (36.3 mg, 0.2 mmol), NaOAc (16.4 mg, 0.2 mmol) and powdered mol­ecular sieves (40 mg, activated 3 Å) were added under air and covered with a septum. The tube was evacuated and refilled with N_2_. Under a positive N_2_ pressure, toluene (2 mL) was added *via* a syringe followed by degassing under a weak vacuum to this tube, and it was refilled with O_2_ three times. The reaction mixture was sealed and stirred at 293 K for 24 h under an O_2_ atmosphere. After completion of the reaction, the solution was cooled to room temperature and diluted with ethyl acetate followed by filtration through a thin pad of Celite. The crude product was purified by flash chromatography (hexa­nes/EtOAc) on silica gel to afford *N*-acetyl benzo[*def*]carbazole **2**. Crystals of the title compound were obtained by thermal evaporation of the pure compound from a 1:1 solution of di­chloro­methane and hexa­nes.

## 6. Refinement

Crystal data, data collection and structure refinement details are summarized in Table 2 ▸. C-bound H atoms were positioned geometrically (C—H = 0.95–0.98 Å) and refined using a riding model, with *U*_iso_(H) = 1.2 or 1.5*U*_eq_(C).

## Supplementary Material

Crystal structure: contains datablock(s) I. DOI: 10.1107/S2056989022004509/ex2054sup1.cif

Structure factors: contains datablock(s) I. DOI: 10.1107/S2056989022004509/ex2054Isup2.hkl

Supporting information file. DOI: 10.1107/S2056989022004509/ex2054Isup3.cml

CCDC reference: 2101657

Additional supporting information:  crystallographic information; 3D view; checkCIF report

## Figures and Tables

**Table 1:** Hydrogen-bond geometry (Å, °) *Cg*6 and *Cg*22 are the centroids of the C25–C30 and C52–C57 rings,respectively.

*D*—H⋯*A*	*D*—H	H⋯*A*	*D*⋯*A*	*D*—H⋯*A*
C12—H12⋯O2^i^	0.95	2.48	3.417 (3)	169
C49—H49⋯O1^ii^	0.95	2.42	3.294 (2)	153
C19—H19⋯*Cg*22^iii^	0.95	2.94	3.652 (2)	132
C33—H33⋯*Cg*6^iii^	0.05	2.96	3.756 (2)	142

Symmetry codes: (i) 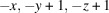 ; (ii) 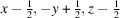 ; (iii) 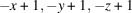 .

**Table 2:** Experimental details

Crystal data	
Chemical formula	C_60_H_38_N_2_O_2_
*M*_r_	818.92
Crystal system, space group	Monoclinic, *P*2_1_/*n*
Temperature (K)	100
*a*, *b*, *c* (Å)	15.835 (2), 7.0408 (8), 37.245 (4)
β (°)	96.464 (4)
*V* (Å^3^)	4126.0 (8)
*Z*	4
Radiation type	Mo *K*α
μ (mm^−1^)	0.08
Crystal size (mm)	0.10 × 0.04 × 0.01

Data collection	
Diffractometer	Bruker APEXII CCD
Absorption correction	Multi-scan (*SADABS*; Bruker, 2013 ▸)
*T*_min_, *T*_max_	0.663, 0.745
No. of measured, independent and observed [*I* > 2σ(*I*)] reflections	24738, 8499, 5655
*R*_int_	0.050
(sin θ/λ)_max_ (Å^−1^)	0.627

Refinement	
*R*[*F* ^2^ > 2σ(*F* ^2^)], *wR*(*F* ^2^), *S*	0.050, 0.113, 1.00
No. of reflections	8499
No. of parameters	579
H-atom treatment	H-atom parameters constrained
Δρ_max_, Δρ_min_ (e Å^−3^)	0.21, −0.21

Computer programs: *APEX2* and *SAINT* (Bruker, 2013 ▸), *SHELXD* and *SHELXTL* (Sheldrick, 2008 ▸) and *SHELXL2014/6* (Sheldrick, 2015 ▸).

## supplementary crystallographic information

### Crystal data

**Table:**

C_60_H_38_N_2_O_2_	*F*(000) = 1712
*M**_r_* = 818.92	*D*_x_ = 1.318 Mg m^−^^3^
Monoclinic, *P*2_1_/*n*	Mo *K*α radiation, λ = 0.71073 Å
*a* = 15.835 (2) Å	Cell parameters from 4269 reflections
*b* = 7.0408 (8) Å	θ = 2.6–26.2°
*c* = 37.245 (4) Å	µ = 0.08 mm^−^^1^
β = 96.464 (4)°	*T* = 100 K
*V* = 4126.0 (8) Å^3^	Lamellar, colorless
*Z* = 4	0.10 × 0.04 × 0.01 mm

### Data collection

**Table:**

Bruker APEXII CCD diffractometer	5655 reflections with *I* > 2σ(*I*)
Detector resolution: 8.3333 pixels mm^-1^	*R*_int_ = 0.050
φ and ω scans	θ_max_ = 26.5°, θ_min_ = 2.6°
Absorption correction: multi-scan (SADABS; Bruker, 2013)	*h* = −19→19
*T*_min_ = 0.663, *T*_max_ = 0.745	*k* = −7→8
24738 measured reflections	*l* = −46→29
8499 independent reflections	

### Refinement

**Table:**

Refinement on *F*^2^	0 restraints
Least-squares matrix: full	Hydrogen site location: inferred from neighbouring sites
*R*[*F*^2^ > 2σ(*F*^2^)] = 0.050	H-atom parameters constrained
*wR*(*F*^2^) = 0.113	*w* = 1/[σ^2^(*F*_o_^2^) + (0.0436*P*)^2^ + 1.1066*P*] where *P* = (*F*_o_^2^ + 2*F*_c_^2^)/3
*S* = 1.00	(Δ/σ)_max_ = 0.001
8499 reflections	Δρ_max_ = 0.21 e Å^−^^3^
579 parameters	Δρ_min_ = −0.21 e Å^−^^3^

### Special details

**Table:**

Geometry. All esds (except the esd in the dihedral angle between two l.s. planes) are estimated using the full covariance matrix. The cell esds are taken into account individually in the estimation of esds in distances, angles and torsion angles; correlations between esds in cell parameters are only used when they are defined by crystal symmetry. An approximate (isotropic) treatment of cell esds is used for estimating esds involving l.s. planes.

### Fractional atomic coordinates and isotropic or equivalent isotropic displacement parameters (Å^2^)

**Table:**

	*x*	*y*	*z*	*U*_iso_*/*U*_eq_	
O1	0.74487 (10)	0.1747 (2)	0.68964 (4)	0.0415 (4)	
O2	0.22802 (9)	0.3551 (2)	0.34700 (4)	0.0362 (4)	
N1	0.65540 (10)	0.1891 (2)	0.63820 (4)	0.0240 (4)	
N2	0.34803 (10)	0.2839 (2)	0.32282 (4)	0.0206 (4)	
C1	0.80629 (14)	0.1151 (4)	0.63534 (6)	0.0419 (6)	
H1A	0.8596	0.1073	0.6514	0.063*	
H1B	0.8110	0.2147	0.6173	0.063*	
H1C	0.7950	−0.0070	0.6231	0.063*	
C2	0.73527 (13)	0.1615 (3)	0.65688 (6)	0.0303 (5)	
C3	0.58209 (13)	0.2340 (3)	0.65598 (5)	0.0224 (4)	
C4	0.51389 (13)	0.2467 (3)	0.62913 (5)	0.0201 (4)	
C5	0.43028 (13)	0.2837 (3)	0.63400 (5)	0.0208 (4)	
C6	0.37150 (12)	0.2849 (2)	0.60078 (5)	0.0186 (4)	
C7	0.28426 (13)	0.3221 (3)	0.60435 (5)	0.0211 (4)	
C8	0.21214 (13)	0.3538 (3)	0.60968 (5)	0.0219 (4)	
C9	0.12705 (12)	0.4010 (3)	0.61566 (5)	0.0210 (4)	
C10	0.05863 (13)	0.3555 (3)	0.59016 (5)	0.0249 (5)	
H10	0.0683	0.2903	0.5687	0.030*	
C11	−0.02299 (14)	0.4048 (3)	0.59603 (5)	0.0308 (5)	
H11	−0.0692	0.3734	0.5785	0.037*	
C12	−0.03801 (14)	0.4993 (3)	0.62720 (6)	0.0316 (5)	
H12	−0.0943	0.5333	0.6311	0.038*	
C13	0.62873 (12)	0.1764 (3)	0.59994 (5)	0.0208 (4)	
C14	0.66663 (12)	0.1343 (3)	0.56942 (5)	0.0235 (4)	
H14	0.7255	0.1056	0.5706	0.028*	
C15	0.61385 (12)	0.1359 (3)	0.53622 (5)	0.0227 (4)	
H15	0.6392	0.1070	0.5149	0.027*	
C16	0.52804 (12)	0.1766 (3)	0.53256 (5)	0.0196 (4)	
H16	0.4963	0.1764	0.5093	0.024*	
C17	0.48803 (12)	0.2183 (2)	0.56351 (5)	0.0167 (4)	
C18	0.39959 (12)	0.2569 (2)	0.56697 (5)	0.0173 (4)	
C19	0.11147 (13)	0.4960 (3)	0.64717 (5)	0.0253 (5)	
H19	0.1573	0.5273	0.6649	0.030*	
C20	0.02936 (14)	0.5442 (3)	0.65260 (6)	0.0305 (5)	
H20	0.0191	0.6089	0.6741	0.037*	
C21	0.41488 (14)	0.3148 (3)	0.67003 (5)	0.0245 (5)	
H21	0.3592	0.3426	0.6758	0.029*	
C22	0.48259 (14)	0.3041 (3)	0.69689 (5)	0.0299 (5)	
H22	0.4711	0.3260	0.7210	0.036*	
C23	0.56690 (14)	0.2634 (3)	0.69129 (5)	0.0287 (5)	
H23	0.6110	0.2564	0.7108	0.034*	
C24	0.54160 (12)	0.2140 (3)	0.59572 (5)	0.0188 (4)	
C25	0.33834 (11)	0.2660 (3)	0.53370 (5)	0.0177 (4)	
C26	0.34917 (12)	0.3975 (3)	0.50668 (5)	0.0202 (4)	
H26	0.3961	0.4823	0.5095	0.024*	
C27	0.29199 (13)	0.4057 (3)	0.47564 (5)	0.0247 (5)	
H27	0.2998	0.4966	0.4574	0.030*	
C28	0.22389 (12)	0.2826 (3)	0.47107 (5)	0.0253 (5)	
H28	0.1848	0.2886	0.4498	0.030*	
C29	0.21288 (12)	0.1506 (3)	0.49758 (5)	0.0251 (5)	
H29	0.1664	0.0647	0.4945	0.030*	
C30	0.26971 (12)	0.1436 (3)	0.52868 (5)	0.0218 (4)	
H30	0.2614	0.0531	0.5469	0.026*	
C31	0.98472 (13)	0.2404 (3)	0.48214 (5)	0.0291 (5)	
H31	1.0332	0.2476	0.4996	0.035*	
C32	0.90969 (13)	0.3266 (3)	0.48863 (5)	0.0263 (5)	
H32	0.9069	0.3943	0.5105	0.032*	
C33	0.83845 (12)	0.3151 (3)	0.46351 (5)	0.0219 (4)	
H33	0.7868	0.3739	0.4682	0.026*	
C34	0.84273 (12)	0.2167 (3)	0.43118 (5)	0.0185 (4)	
C35	0.76930 (12)	0.2040 (3)	0.40509 (5)	0.0189 (4)	
C36	0.70893 (12)	0.1952 (3)	0.38286 (5)	0.0185 (4)	
C37	0.63290 (12)	0.1977 (2)	0.35821 (5)	0.0175 (4)	
C38	0.55469 (12)	0.2330 (2)	0.37392 (5)	0.0179 (4)	
C39	0.48281 (12)	0.2459 (2)	0.34919 (5)	0.0177 (4)	
C40	0.40077 (12)	0.2841 (3)	0.35681 (5)	0.0196 (4)	
C41	0.26149 (13)	0.3182 (3)	0.32008 (5)	0.0254 (5)	
C42	0.21216 (13)	0.3046 (3)	0.28353 (5)	0.0331 (5)	
H42A	0.1520	0.3293	0.2855	0.050*	
H42B	0.2186	0.1770	0.2737	0.050*	
H42C	0.2336	0.3987	0.2674	0.050*	
C43	0.98923 (13)	0.1438 (3)	0.45019 (5)	0.0283 (5)	
H43	1.0409	0.0847	0.4457	0.034*	
C44	0.91876 (12)	0.1324 (3)	0.42466 (5)	0.0237 (5)	
H44	0.9224	0.0667	0.4026	0.028*	
C45	0.63535 (12)	0.1767 (2)	0.32106 (5)	0.0176 (4)	
C46	0.55753 (12)	0.1896 (2)	0.29627 (5)	0.0175 (4)	
C47	0.54453 (12)	0.1717 (3)	0.25823 (5)	0.0214 (4)	
H47	0.5908	0.1441	0.2449	0.026*	
C48	0.46346 (12)	0.1948 (3)	0.24062 (5)	0.0232 (4)	
H48	0.4565	0.1837	0.2150	0.028*	
C49	0.39071 (12)	0.2335 (3)	0.25765 (5)	0.0224 (4)	
H49	0.3365	0.2495	0.2442	0.027*	
C50	0.40171 (12)	0.2472 (3)	0.29484 (5)	0.0196 (4)	
C51	0.48426 (12)	0.2248 (2)	0.31221 (5)	0.0181 (4)	
C52	0.71793 (12)	0.1450 (3)	0.30673 (5)	0.0184 (4)	
C53	0.74376 (12)	0.2607 (3)	0.27948 (5)	0.0213 (4)	
H53	0.7066	0.3564	0.2689	0.026*	
C54	0.82284 (12)	0.2372 (3)	0.26773 (5)	0.0220 (4)	
H54	0.8395	0.3167	0.2492	0.026*	
C55	0.87774 (12)	0.0993 (3)	0.28276 (5)	0.0232 (4)	
H55	0.9325	0.0852	0.2749	0.028*	
C56	0.85243 (12)	−0.0183 (3)	0.30930 (5)	0.0227 (5)	
H56	0.8897	−0.1148	0.3195	0.027*	
C57	0.77323 (12)	0.0036 (3)	0.32107 (5)	0.0201 (4)	
H57	0.7564	−0.0788	0.3392	0.024*	
C58	0.54071 (12)	0.2608 (3)	0.41017 (5)	0.0209 (4)	
H58	0.5867	0.2545	0.4289	0.025*	
C59	0.45910 (13)	0.2973 (3)	0.41824 (5)	0.0225 (4)	
H59	0.4510	0.3150	0.4429	0.027*	
C60	0.38710 (13)	0.3102 (3)	0.39243 (5)	0.0227 (4)	
H60	0.3321	0.3354	0.3992	0.027*	

### Atomic displacement parameters (Å^2^)

**Table:**

	*U*^11^	*U*^22^	*U*^33^	*U*^12^	*U*^13^	*U*^23^
O1	0.0442 (10)	0.0477 (10)	0.0288 (9)	−0.0090 (8)	−0.0134 (7)	0.0041 (7)
O2	0.0260 (8)	0.0502 (10)	0.0333 (9)	0.0050 (7)	0.0069 (7)	−0.0010 (7)
N1	0.0293 (10)	0.0201 (9)	0.0207 (8)	−0.0037 (7)	−0.0054 (7)	0.0012 (7)
N2	0.0204 (9)	0.0200 (9)	0.0208 (8)	−0.0013 (7)	0.0002 (7)	0.0002 (7)
C1	0.0284 (13)	0.0526 (16)	0.0417 (14)	−0.0024 (11)	−0.0091 (11)	0.0081 (11)
C2	0.0311 (12)	0.0246 (12)	0.0319 (12)	−0.0068 (9)	−0.0109 (10)	0.0046 (9)
C3	0.0301 (11)	0.0140 (10)	0.0224 (10)	−0.0064 (9)	−0.0003 (9)	0.0014 (8)
C4	0.0327 (12)	0.0111 (10)	0.0162 (9)	−0.0051 (8)	0.0011 (8)	0.0002 (7)
C5	0.0335 (12)	0.0105 (9)	0.0190 (10)	−0.0055 (8)	0.0051 (8)	0.0006 (7)
C6	0.0263 (11)	0.0092 (9)	0.0209 (10)	−0.0028 (8)	0.0047 (8)	0.0008 (7)
C7	0.0324 (12)	0.0138 (10)	0.0176 (10)	−0.0010 (9)	0.0053 (9)	0.0016 (7)
C8	0.0335 (12)	0.0127 (10)	0.0201 (10)	−0.0024 (9)	0.0062 (9)	0.0029 (8)
C9	0.0286 (11)	0.0148 (10)	0.0209 (10)	−0.0008 (8)	0.0086 (9)	0.0038 (8)
C10	0.0349 (12)	0.0189 (11)	0.0215 (10)	−0.0031 (9)	0.0059 (9)	−0.0026 (8)
C11	0.0307 (12)	0.0308 (12)	0.0305 (12)	−0.0039 (10)	0.0018 (10)	0.0014 (9)
C12	0.0257 (12)	0.0291 (13)	0.0418 (13)	−0.0011 (10)	0.0116 (10)	−0.0002 (10)
C13	0.0281 (11)	0.0119 (10)	0.0210 (10)	−0.0047 (8)	−0.0026 (8)	0.0006 (8)
C14	0.0208 (11)	0.0201 (11)	0.0292 (11)	−0.0014 (8)	0.0014 (9)	0.0006 (8)
C15	0.0278 (11)	0.0198 (11)	0.0214 (10)	−0.0014 (9)	0.0059 (9)	−0.0010 (8)
C16	0.0249 (11)	0.0160 (10)	0.0174 (9)	−0.0035 (8)	0.0003 (8)	0.0010 (7)
C17	0.0245 (10)	0.0093 (9)	0.0161 (9)	−0.0025 (8)	0.0022 (8)	0.0003 (7)
C18	0.0237 (10)	0.0093 (9)	0.0190 (9)	−0.0025 (8)	0.0025 (8)	0.0015 (7)
C19	0.0319 (12)	0.0237 (11)	0.0212 (10)	−0.0050 (9)	0.0072 (9)	−0.0024 (8)
C20	0.0368 (13)	0.0281 (12)	0.0294 (11)	−0.0044 (10)	0.0155 (10)	−0.0078 (9)
C21	0.0398 (13)	0.0161 (11)	0.0189 (10)	−0.0057 (9)	0.0087 (9)	−0.0009 (8)
C22	0.0514 (15)	0.0230 (12)	0.0161 (10)	−0.0101 (10)	0.0066 (10)	−0.0016 (8)
C23	0.0476 (14)	0.0197 (11)	0.0170 (10)	−0.0106 (10)	−0.0045 (9)	0.0002 (8)
C24	0.0256 (11)	0.0116 (9)	0.0191 (9)	−0.0039 (8)	0.0021 (8)	−0.0007 (7)
C25	0.0200 (10)	0.0163 (10)	0.0173 (9)	0.0027 (8)	0.0048 (8)	−0.0004 (7)
C26	0.0229 (11)	0.0180 (10)	0.0200 (10)	−0.0017 (8)	0.0044 (8)	−0.0008 (8)
C27	0.0318 (12)	0.0230 (11)	0.0199 (10)	0.0038 (9)	0.0061 (9)	0.0034 (8)
C28	0.0248 (11)	0.0266 (12)	0.0235 (10)	0.0059 (9)	−0.0014 (9)	−0.0001 (9)
C29	0.0204 (11)	0.0239 (11)	0.0304 (11)	−0.0002 (9)	−0.0001 (9)	0.0000 (9)
C30	0.0237 (11)	0.0191 (11)	0.0233 (10)	−0.0020 (8)	0.0056 (8)	0.0032 (8)
C31	0.0277 (12)	0.0270 (12)	0.0290 (11)	−0.0037 (10)	−0.0126 (9)	0.0031 (9)
C32	0.0361 (13)	0.0231 (11)	0.0185 (10)	−0.0037 (9)	−0.0018 (9)	−0.0025 (8)
C33	0.0248 (11)	0.0193 (11)	0.0212 (10)	−0.0018 (8)	0.0006 (8)	0.0007 (8)
C34	0.0229 (10)	0.0128 (10)	0.0190 (9)	−0.0033 (8)	−0.0012 (8)	0.0040 (7)
C35	0.0236 (11)	0.0142 (10)	0.0184 (10)	−0.0023 (8)	0.0005 (8)	0.0021 (7)
C36	0.0251 (11)	0.0132 (10)	0.0174 (9)	−0.0020 (8)	0.0030 (9)	0.0011 (7)
C37	0.0228 (10)	0.0101 (9)	0.0183 (9)	−0.0020 (8)	−0.0029 (8)	0.0024 (7)
C38	0.0242 (10)	0.0097 (9)	0.0190 (9)	−0.0037 (8)	−0.0011 (8)	0.0009 (7)
C39	0.0241 (11)	0.0120 (10)	0.0170 (9)	−0.0019 (8)	0.0021 (8)	0.0005 (7)
C40	0.0241 (11)	0.0126 (10)	0.0211 (10)	−0.0039 (8)	−0.0010 (8)	0.0001 (8)
C41	0.0234 (11)	0.0207 (11)	0.0317 (11)	0.0011 (9)	0.0017 (9)	0.0029 (9)
C42	0.0226 (11)	0.0424 (14)	0.0328 (12)	0.0048 (10)	−0.0031 (9)	0.0055 (10)
C43	0.0224 (11)	0.0247 (12)	0.0362 (12)	0.0028 (9)	−0.0034 (9)	−0.0006 (9)
C44	0.0297 (12)	0.0167 (11)	0.0236 (10)	−0.0021 (9)	−0.0015 (9)	−0.0016 (8)
C45	0.0217 (10)	0.0104 (9)	0.0199 (9)	−0.0020 (8)	−0.0017 (8)	0.0015 (7)
C46	0.0227 (10)	0.0112 (9)	0.0178 (9)	−0.0021 (8)	−0.0009 (8)	0.0004 (7)
C47	0.0251 (11)	0.0192 (10)	0.0195 (10)	0.0000 (8)	0.0004 (8)	−0.0002 (8)
C48	0.0297 (12)	0.0233 (11)	0.0155 (9)	−0.0015 (9)	−0.0025 (8)	−0.0012 (8)
C49	0.0237 (11)	0.0207 (11)	0.0211 (10)	−0.0012 (9)	−0.0056 (8)	0.0012 (8)
C50	0.0221 (10)	0.0137 (10)	0.0225 (10)	−0.0010 (8)	0.0008 (8)	−0.0007 (8)
C51	0.0227 (10)	0.0117 (9)	0.0188 (10)	−0.0031 (8)	−0.0024 (8)	0.0001 (7)
C52	0.0230 (10)	0.0153 (10)	0.0160 (9)	−0.0026 (8)	−0.0019 (8)	−0.0018 (7)
C53	0.0277 (11)	0.0176 (10)	0.0174 (9)	−0.0003 (9)	−0.0033 (8)	0.0012 (8)
C54	0.0303 (11)	0.0201 (11)	0.0152 (9)	−0.0056 (9)	0.0014 (8)	0.0013 (8)
C55	0.0234 (11)	0.0268 (11)	0.0195 (10)	−0.0016 (9)	0.0035 (8)	−0.0025 (8)
C56	0.0258 (11)	0.0210 (11)	0.0201 (10)	0.0053 (9)	−0.0024 (9)	0.0003 (8)
C57	0.0260 (11)	0.0168 (10)	0.0171 (10)	−0.0021 (8)	0.0000 (8)	0.0017 (7)
C58	0.0296 (11)	0.0149 (10)	0.0177 (10)	−0.0038 (8)	0.0002 (8)	0.0010 (7)
C59	0.0337 (12)	0.0179 (10)	0.0167 (9)	−0.0047 (9)	0.0052 (9)	0.0011 (8)
C60	0.0266 (11)	0.0167 (11)	0.0256 (11)	−0.0037 (8)	0.0064 (9)	−0.0011 (8)

### Geometric parameters (Å, º)

**Table:**

O1—C2	1.216 (2)	C28—H28	0.9500
O2—C41	1.214 (2)	C29—C30	1.386 (3)
N1—C2	1.387 (3)	C29—H29	0.9500
N1—C3	1.435 (3)	C30—H30	0.9500
N1—C13	1.443 (2)	C31—C32	1.379 (3)
N2—C41	1.384 (2)	C31—C43	1.380 (3)
N2—C40	1.436 (2)	C31—H31	0.9500
N2—C50	1.440 (2)	C32—C33	1.385 (3)
C1—C2	1.489 (3)	C32—H32	0.9500
C1—H1A	0.9800	C33—C34	1.397 (2)
C1—H1B	0.9800	C33—H33	0.9500
C1—H1C	0.9800	C34—C44	1.388 (3)
C3—C23	1.379 (3)	C34—C35	1.432 (3)
C3—C4	1.390 (3)	C35—C36	1.194 (2)
C4—C5	1.381 (3)	C36—C37	1.430 (3)
C4—C24	1.385 (3)	C37—C45	1.396 (2)
C5—C21	1.408 (2)	C37—C38	1.450 (3)
C5—C6	1.462 (3)	C38—C39	1.384 (2)
C6—C18	1.396 (2)	C38—C58	1.406 (2)
C6—C7	1.427 (3)	C39—C40	1.387 (3)
C7—C8	1.202 (3)	C39—C51	1.388 (2)
C8—C9	1.429 (3)	C40—C60	1.380 (3)
C9—C10	1.395 (3)	C41—C42	1.494 (3)
C9—C19	1.396 (3)	C42—H42A	0.9800
C10—C11	1.379 (3)	C42—H42B	0.9800
C10—H10	0.9500	C42—H42C	0.9800
C11—C12	1.382 (3)	C43—C44	1.384 (3)
C11—H11	0.9500	C43—H43	0.9500
C12—C20	1.380 (3)	C44—H44	0.9500
C12—H12	0.9500	C45—C46	1.457 (2)
C13—C14	1.376 (3)	C45—C52	1.484 (3)
C13—C24	1.396 (3)	C46—C51	1.384 (3)
C14—C15	1.412 (3)	C46—C47	1.414 (2)
C14—H14	0.9500	C47—C48	1.384 (3)
C15—C16	1.380 (3)	C47—H47	0.9500
C15—H15	0.9500	C48—C49	1.403 (3)
C16—C17	1.407 (2)	C48—H48	0.9500
C16—H16	0.9500	C49—C50	1.380 (2)
C17—C24	1.390 (2)	C49—H49	0.9500
C17—C18	1.447 (3)	C50—C51	1.401 (3)
C18—C25	1.487 (2)	C52—C57	1.392 (2)
C19—C20	1.380 (3)	C52—C53	1.398 (3)
C19—H19	0.9500	C53—C54	1.382 (3)
C20—H20	0.9500	C53—H53	0.9500
C21—C22	1.384 (3)	C54—C55	1.379 (3)
C21—H21	0.9500	C54—H54	0.9500
C22—C23	1.404 (3)	C55—C56	1.383 (3)
C22—H22	0.9500	C55—H55	0.9500
C23—H23	0.9500	C56—C57	1.383 (3)
C25—C30	1.383 (3)	C56—H56	0.9500
C25—C26	1.392 (2)	C57—H57	0.9500
C26—C27	1.387 (2)	C58—C59	1.384 (3)
C26—H26	0.9500	C58—H58	0.9500
C27—C28	1.379 (3)	C59—C60	1.409 (3)
C27—H27	0.9500	C59—H59	0.9500
C28—C29	1.382 (3)	C60—H60	0.9500
			
C2—N1—C3	122.60 (16)	C25—C30—C29	121.13 (18)
C2—N1—C13	129.48 (17)	C25—C30—H30	119.4
C3—N1—C13	107.90 (15)	C29—C30—H30	119.4
C41—N2—C40	122.53 (16)	C32—C31—C43	119.82 (18)
C41—N2—C50	129.64 (15)	C32—C31—H31	120.1
C40—N2—C50	107.81 (14)	C43—C31—H31	120.1
C2—C1—H1A	109.5	C31—C32—C33	120.55 (18)
C2—C1—H1B	109.5	C31—C32—H32	119.7
H1A—C1—H1B	109.5	C33—C32—H32	119.7
C2—C1—H1C	109.5	C32—C33—C34	119.76 (19)
H1A—C1—H1C	109.5	C32—C33—H33	120.1
H1B—C1—H1C	109.5	C34—C33—H33	120.1
O1—C2—N1	119.8 (2)	C44—C34—C33	119.34 (17)
O1—C2—C1	122.72 (19)	C44—C34—C35	120.57 (17)
N1—C2—C1	117.51 (18)	C33—C34—C35	120.09 (17)
C23—C3—C4	118.14 (19)	C36—C35—C34	178.7 (2)
C23—C3—N1	135.25 (18)	C35—C36—C37	174.6 (2)
C4—C3—N1	106.60 (16)	C45—C37—C36	121.32 (17)
C5—C4—C24	123.70 (17)	C45—C37—C38	122.58 (16)
C5—C4—C3	126.63 (17)	C36—C37—C38	116.03 (15)
C24—C4—C3	109.67 (18)	C39—C38—C58	115.03 (17)
C4—C5—C21	115.21 (17)	C39—C38—C37	114.75 (16)
C4—C5—C6	114.73 (16)	C58—C38—C37	130.20 (16)
C21—C5—C6	130.06 (19)	C38—C39—C40	126.57 (17)
C18—C6—C7	121.34 (16)	C38—C39—C51	123.29 (18)
C18—C6—C5	121.69 (17)	C40—C39—C51	110.13 (16)
C7—C6—C5	116.94 (16)	C60—C40—C39	118.30 (17)
C8—C7—C6	175.84 (19)	C60—C40—N2	135.14 (18)
C7—C8—C9	177.2 (2)	C39—C40—N2	106.55 (15)
C10—C9—C19	119.00 (18)	O2—C41—N2	119.81 (18)
C10—C9—C8	120.92 (17)	O2—C41—C42	122.28 (18)
C19—C9—C8	120.07 (18)	N2—C41—C42	117.89 (18)
C11—C10—C9	120.27 (18)	C41—C42—H42A	109.5
C11—C10—H10	119.9	C41—C42—H42B	109.5
C9—C10—H10	119.9	H42A—C42—H42B	109.5
C10—C11—C12	120.49 (19)	C41—C42—H42C	109.5
C10—C11—H11	119.8	H42A—C42—H42C	109.5
C12—C11—H11	119.8	H42B—C42—H42C	109.5
C20—C12—C11	119.5 (2)	C31—C43—C44	120.33 (19)
C20—C12—H12	120.2	C31—C43—H43	119.8
C11—C12—H12	120.2	C44—C43—H43	119.8
C14—C13—C24	117.72 (17)	C43—C44—C34	120.20 (18)
C14—C13—N1	136.29 (18)	C43—C44—H44	119.9
C24—C13—N1	105.97 (16)	C34—C44—H44	119.9
C13—C14—C15	116.82 (18)	C37—C45—C46	120.36 (17)
C13—C14—H14	121.6	C37—C45—C52	119.74 (16)
C15—C14—H14	121.6	C46—C45—C52	119.89 (15)
C16—C15—C14	124.55 (18)	C51—C46—C47	114.29 (16)
C16—C15—H15	117.7	C51—C46—C45	115.42 (15)
C14—C15—H15	117.7	C47—C46—C45	130.29 (17)
C15—C16—C17	119.42 (17)	C48—C47—C46	119.14 (18)
C15—C16—H16	120.3	C48—C47—H47	120.4
C17—C16—H16	120.3	C46—C47—H47	120.4
C24—C17—C16	114.54 (17)	C47—C48—C49	125.01 (17)
C24—C17—C18	115.48 (16)	C47—C48—H48	117.5
C16—C17—C18	129.93 (16)	C49—C48—H48	117.5
C6—C18—C17	121.13 (16)	C50—C49—C48	116.75 (17)
C6—C18—C25	120.12 (17)	C50—C49—H49	121.6
C17—C18—C25	118.75 (15)	C48—C49—H49	121.6
C20—C19—C9	119.95 (19)	C49—C50—C51	117.50 (17)
C20—C19—H19	120.0	C49—C50—N2	136.11 (17)
C9—C19—H19	120.0	C51—C50—N2	106.38 (15)
C19—C20—C12	120.75 (19)	C46—C51—C39	123.59 (16)
C19—C20—H20	119.6	C46—C51—C50	127.29 (16)
C12—C20—H20	119.6	C39—C51—C50	109.12 (17)
C22—C21—C5	118.5 (2)	C57—C52—C53	118.19 (18)
C22—C21—H21	120.7	C57—C52—C45	120.77 (16)
C5—C21—H21	120.7	C53—C52—C45	121.00 (16)
C21—C22—C23	125.18 (18)	C54—C53—C52	120.64 (17)
C21—C22—H22	117.4	C54—C53—H53	119.7
C23—C22—H22	117.4	C52—C53—H53	119.7
C3—C23—C22	116.31 (18)	C55—C54—C53	120.53 (17)
C3—C23—H23	121.8	C55—C54—H54	119.7
C22—C23—H23	121.8	C53—C54—H54	119.7
C4—C24—C17	123.23 (18)	C54—C55—C56	119.44 (18)
C4—C24—C13	109.85 (16)	C54—C55—H55	120.3
C17—C24—C13	126.92 (17)	C56—C55—H55	120.3
C30—C25—C26	118.41 (16)	C57—C56—C55	120.37 (18)
C30—C25—C18	120.97 (16)	C57—C56—H56	119.8
C26—C25—C18	120.62 (16)	C55—C56—H56	119.8
C27—C26—C25	120.55 (18)	C56—C57—C52	120.80 (17)
C27—C26—H26	119.7	C56—C57—H57	119.6
C25—C26—H26	119.7	C52—C57—H57	119.6
C28—C27—C26	120.31 (18)	C59—C58—C38	119.11 (17)
C28—C27—H27	119.8	C59—C58—H58	120.4
C26—C27—H27	119.8	C38—C58—H58	120.4
C27—C28—C29	119.63 (18)	C58—C59—C60	124.57 (17)
C27—C28—H28	120.2	C58—C59—H59	117.7
C29—C28—H28	120.2	C60—C59—H59	117.7
C28—C29—C30	119.96 (19)	C40—C60—C59	116.42 (18)
C28—C29—H29	120.0	C40—C60—H60	121.8
C30—C29—H29	120.0	C59—C60—H60	121.8
			
C3—N1—C2—O1	0.2 (3)	C43—C31—C32—C33	0.7 (3)
C13—N1—C2—O1	−178.38 (18)	C31—C32—C33—C34	−0.5 (3)
C3—N1—C2—C1	179.85 (18)	C32—C33—C34—C44	−0.3 (3)
C13—N1—C2—C1	1.3 (3)	C32—C33—C34—C35	179.92 (17)
C2—N1—C3—C23	1.0 (3)	C45—C37—C38—C39	−0.5 (3)
C13—N1—C3—C23	179.8 (2)	C36—C37—C38—C39	176.31 (16)
C2—N1—C3—C4	−178.59 (17)	C45—C37—C38—C58	−178.77 (18)
C13—N1—C3—C4	0.2 (2)	C36—C37—C38—C58	−1.9 (3)
C23—C3—C4—C5	−1.1 (3)	C58—C38—C39—C40	0.5 (3)
N1—C3—C4—C5	178.62 (17)	C37—C38—C39—C40	−178.01 (17)
C23—C3—C4—C24	179.72 (17)	C58—C38—C39—C51	179.25 (17)
N1—C3—C4—C24	−0.6 (2)	C37—C38—C39—C51	0.7 (3)
C24—C4—C5—C21	−179.48 (17)	C38—C39—C40—C60	−0.9 (3)
C3—C4—C5—C21	1.4 (3)	C51—C39—C40—C60	−179.79 (17)
C24—C4—C5—C6	0.3 (3)	C38—C39—C40—N2	179.81 (17)
C3—C4—C5—C6	−178.78 (18)	C51—C39—C40—N2	0.9 (2)
C4—C5—C6—C18	−2.1 (3)	C41—N2—C40—C60	1.2 (3)
C21—C5—C6—C18	177.73 (18)	C50—N2—C40—C60	−180.0 (2)
C4—C5—C6—C7	179.89 (16)	C41—N2—C40—C39	−179.69 (16)
C21—C5—C6—C7	−0.3 (3)	C50—N2—C40—C39	−0.82 (19)
C19—C9—C10—C11	0.4 (3)	C40—N2—C41—O2	2.0 (3)
C8—C9—C10—C11	−178.91 (18)	C50—N2—C41—O2	−176.59 (18)
C9—C10—C11—C12	−0.1 (3)	C40—N2—C41—C42	−176.92 (17)
C10—C11—C12—C20	−0.1 (3)	C50—N2—C41—C42	4.5 (3)
C2—N1—C13—C14	0.8 (4)	C32—C31—C43—C44	−0.2 (3)
C3—N1—C13—C14	−177.9 (2)	C31—C43—C44—C34	−0.6 (3)
C2—N1—C13—C24	178.92 (18)	C33—C34—C44—C43	0.8 (3)
C3—N1—C13—C24	0.21 (19)	C35—C34—C44—C43	−179.37 (18)
C24—C13—C14—C15	1.0 (3)	C36—C37—C45—C46	−176.79 (16)
N1—C13—C14—C15	178.96 (19)	C38—C37—C45—C46	−0.1 (3)
C13—C14—C15—C16	0.1 (3)	C36—C37—C45—C52	2.0 (3)
C14—C15—C16—C17	−0.6 (3)	C38—C37—C45—C52	178.67 (16)
C15—C16—C17—C24	0.0 (3)	C37—C45—C46—C51	0.6 (2)
C15—C16—C17—C18	−177.04 (18)	C52—C45—C46—C51	−178.20 (16)
C7—C6—C18—C17	−179.69 (17)	C37—C45—C46—C47	−179.38 (18)
C5—C6—C18—C17	2.3 (3)	C52—C45—C46—C47	1.9 (3)
C7—C6—C18—C25	0.2 (3)	C51—C46—C47—C48	1.7 (3)
C5—C6—C18—C25	−177.82 (16)	C45—C46—C47—C48	−178.41 (18)
C24—C17—C18—C6	−0.8 (2)	C46—C47—C48—C49	−0.8 (3)
C16—C17—C18—C6	176.27 (18)	C47—C48—C49—C50	−0.7 (3)
C24—C17—C18—C25	179.37 (16)	C48—C49—C50—C51	1.1 (3)
C16—C17—C18—C25	−3.6 (3)	C48—C49—C50—N2	179.7 (2)
C10—C9—C19—C20	−0.3 (3)	C41—N2—C50—C49	0.5 (4)
C8—C9—C19—C20	178.92 (18)	C40—N2—C50—C49	−178.3 (2)
C9—C19—C20—C12	0.1 (3)	C41—N2—C50—C51	179.20 (18)
C11—C12—C20—C19	0.1 (3)	C40—N2—C50—C51	0.44 (19)
C4—C5—C21—C22	−0.7 (3)	C47—C46—C51—C39	179.56 (17)
C6—C5—C21—C22	179.52 (18)	C45—C46—C51—C39	−0.4 (3)
C5—C21—C22—C23	−0.3 (3)	C47—C46—C51—C50	−1.3 (3)
C4—C3—C23—C22	0.0 (3)	C45—C46—C51—C50	178.79 (18)
N1—C3—C23—C22	−179.6 (2)	C38—C39—C51—C46	−0.3 (3)
C21—C22—C23—C3	0.6 (3)	C40—C39—C51—C46	178.65 (17)
C5—C4—C24—C17	1.2 (3)	C38—C39—C51—C50	−179.60 (17)
C3—C4—C24—C17	−179.56 (17)	C40—C39—C51—C50	−0.7 (2)
C5—C4—C24—C13	−178.49 (17)	C49—C50—C51—C46	−0.1 (3)
C3—C4—C24—C13	0.8 (2)	N2—C50—C51—C46	−179.15 (17)
C16—C17—C24—C4	−178.47 (17)	C49—C50—C51—C39	179.12 (16)
C18—C17—C24—C4	−1.0 (3)	N2—C50—C51—C39	0.1 (2)
C16—C17—C24—C13	1.2 (3)	C37—C45—C52—C57	50.8 (2)
C18—C17—C24—C13	178.67 (17)	C46—C45—C52—C57	−130.45 (18)
C14—C13—C24—C4	177.94 (17)	C37—C45—C52—C53	−126.75 (19)
N1—C13—C24—C4	−0.6 (2)	C46—C45—C52—C53	52.0 (2)
C14—C13—C24—C17	−1.7 (3)	C57—C52—C53—C54	−1.3 (3)
N1—C13—C24—C17	179.75 (17)	C45—C52—C53—C54	176.27 (16)
C6—C18—C25—C30	−59.7 (2)	C52—C53—C54—C55	−0.1 (3)
C17—C18—C25—C30	120.12 (19)	C53—C54—C55—C56	1.2 (3)
C6—C18—C25—C26	120.7 (2)	C54—C55—C56—C57	−0.9 (3)
C17—C18—C25—C26	−59.4 (2)	C55—C56—C57—C52	−0.6 (3)
C30—C25—C26—C27	0.4 (3)	C53—C52—C57—C56	1.7 (3)
C18—C25—C26—C27	179.97 (17)	C45—C52—C57—C56	−175.93 (17)
C25—C26—C27—C28	−0.4 (3)	C39—C38—C58—C59	0.1 (2)
C26—C27—C28—C29	−0.1 (3)	C37—C38—C58—C59	178.33 (18)
C27—C28—C29—C30	0.6 (3)	C38—C58—C59—C60	−0.3 (3)
C26—C25—C30—C29	0.1 (3)	C39—C40—C60—C59	0.6 (3)
C18—C25—C30—C29	−179.48 (17)	N2—C40—C60—C59	179.69 (19)
C28—C29—C30—C25	−0.6 (3)	C58—C59—C60—C40	−0.1 (3)

### Hydrogen-bond geometry (Å, º)

*Cg*6 and *Cg*22 are the centroids of the C25–C30 and C52–C57 rings,respectively.

**Table:**

*D*—H···*A*	*D*—H	H···*A*	*D*···*A*	*D*—H···*A*
C12—H12···O2^i^	0.95	2.48	3.417 (3)	169
C49—H49···O1^ii^	0.95	2.42	3.294 (2)	153
C19—H19···*Cg*22^iii^	0.95	2.94	3.652 (2)	132
C33—H33···*Cg*6^iii^	0.05	2.96	3.756 (2)	142

Symmetry codes: (i) −*x*, −*y*+1, −*z*+1; (ii) *x*−1/2, −*y*+1/2, *z*−1/2; (iii) −*x*+1, −*y*+1, −*z*+1.
